# Acid ceramidase involved in pathogenic cascade leading to accumulation of α-synuclein in iPSC model of *GBA1*-associated Parkinson’s disease

**DOI:** 10.1093/hmg/ddad025

**Published:** 2023-02-08

**Authors:** Manoj Kumar, Manasa P Srikanth, Michela Deleidi, Penelope J Hallett, Ole Isacson, Ricardo A Feldman

**Affiliations:** Department of Microbiology and Immunology, University of Maryland School of Medicine, Baltimore, MD 21201, USA; Department of Microbiology and Immunology, University of Maryland School of Medicine, Baltimore, MD 21201, USA; German Center for Neurodegenerative Diseases (DZNE), Tübingen 72076, Germany; Neuroregeneration Research Institute, Harvard Medical School/McLean Hospital, Belmont, MA 02478, USA; Neuroregeneration Research Institute, Harvard Medical School/McLean Hospital, Belmont, MA 02478, USA; Department of Microbiology and Immunology, University of Maryland School of Medicine, Baltimore, MD 21201, USA

## Abstract

Bi-allelic mutations in *GBA1*, the gene that encodes β-glucocerebrosidase (GCase), cause Gaucher disease (GD), whereas mono-allelic mutations do not cause overt pathology. Yet mono- or bi-allelic *GBA1* mutations are the highest known risk factor for Parkinson’s disease (PD). GCase deficiency results in the accumulation of glucosylceramide (GluCer) and its deacylated metabolite glucosylsphingosine (GluSph). Brains from patients with neuronopathic GD have high levels of GluSph, and elevation of this lipid in *GBA1*-associated PD has been reported. To uncover the mechanisms involved in *GBA1*-associated PD, we used human induced pluripotent stem cell-derived dopaminergic (DA) neurons from patients harboring heterozygote mutations in *GBA1* (*GBA1*/PD–DA neurons). We found that compared with gene-edited isogenic controls, *GBA1*/PD–DA neurons exhibit mammalian target of rapamycin complex 1 (mTORC1) hyperactivity, a block in autophagy, an increase in the levels of phosphorylated α-synuclein (129) and α-synuclein aggregation. These alterations were prevented by incubation with mTOR inhibitors. Inhibition of acid ceramidase, the lysosomal enzyme that deacylates GluCer to GluSph, prevented mTOR hyperactivity, restored autophagic flux and lowered α-synuclein levels, suggesting that GluSph was responsible for these alterations. Incubation of gene-edited wild type (WT) controls with exogenous GluSph recapitulated the mTOR/α-synuclein abnormalities of *GBA1*/PD neurons, and these phenotypic alterations were prevented when GluSph treatment was in the presence of mTOR inhibitors. We conclude that GluSph causes an aberrant activation of mTORC1, suppressing normal lysosomal functions, including the clearance of pathogenic α-synuclein species. Our results implicate acid ceramidase in the pathogenesis of *GBA1*-associated PD, suggesting that this enzyme is a potential therapeutic target for treating synucleinopathies caused by GCase deficiency.

## Graphical abstract

**Figure:**
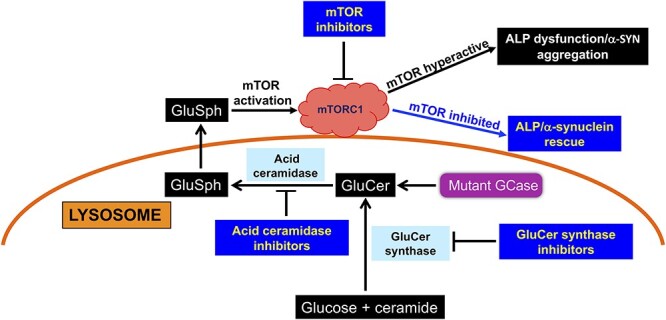

## Introduction

Mutations in enzymes of sphingolipid metabolism are the cause of ˃50 lysosomal storage disorders often associated with neurodegeneration, underlining the importance of sphingolipid balance for maintaining neuronal health (1–5). Bi-allelic mutations in *GBA1*, the gene that encodes lysosomal β-glucocerebrosidase (GCase) cause Gaucher disease (GD), one of the most frequent lysosomal storage diseases. Mild mutations in *GBA1* cause type 1 GD, which affects visceral organs including liver, spleen and bone marrow. Severe *GBA1* mutations cause types 2 and 3 neuronopathic GD (nGD), where in addition to visceral organ involvement, there is fatal neurodegeneration (6–11). Although GD is a recessive disease and carriers of *GBA1* mutations do not exhibit clinical symptoms, individuals with mono- and bi-allelic *GBA1* mutations are at similarly increased risk for development of Parkinson’s disease (PD) (12–17). Mutations in *GBA1* increase the risk of PD by 5- to 20-fold, and ⁓7% of patients with PD harbor *GBA1* mutations. Although the risk of *GBA1*-associated PD and Lewy Body Dementia is still small, this risk increases significantly with age (18,19). PD is characterized by the loss of dopaminergic neurons in the substantia nigra pars compacta, resulting in progressive motor and non-motor clinical manifestations (18,20,21).

GCase hydrolyzes the glycosidic bond in glucosylceramide (GluCer) to yield glucose and ceramide. In type 1 GD, there is an accumulation of GluCer and that of its metabolite glucosylsphingosine (GluSph) in visceral organs. In nGD, glucosylsphingolipids (GSLs) also accumulate in the central nervous system (12). As opposed to GluCer, which is a building block for the synthesis of complex and essential GSLs, no metabolic functions of GluSph have been described and the synthesis of GluSph from glucose and sphingosine is unlikely. Rather, the majority of the elevated GluSph in GD is generated by deacylation of GluCer through the action of acid ceramidase, a lysosomal enzyme that normally hydrolyzes ceramide to sphingosine and fatty acids (22–27). GluSph is almost undetectable in normal cells, but it is highly elevated in tissues and plasma of GD patients. In nGD brains there is up to a 500-fold elevation of GluSph, suggesting that this lipid may play a role in *GBA1*-associated neurodegeneration (10,11,24)**.**

GCase deficiency has been reported to interfere with multiple cellular processes. These include deregulation of the autophagy/lysosomal pathway (ALP), which is essential for neuronal survival. Lysosomes remove damaged organelles, recycle nutrients through autophagy, clear α-synuclein aggregates and regulate vital anabolic and catabolic functions (28,29). Other deleterious effects of GCase deficiency include interference with intracellular protein and vesicle trafficking, mitochondrial dysfunction, alteration of calcium homeostasis (30–32) and downregulation of the Wnt/β-catenin developmental pathway (33–35). However, the mechanisms by which GSL imbalance causes these pathogenic alterations are complex, and still not understood.

The abnormal accumulation of α-synuclein aggregates in midbrain dopaminergic (DA) neurons is a hallmark of PD. A central question in *GBA1*-associated PD is how loss of GCase activity predisposes DA neurons to the accumulation of α-synuclein. It has been reported that GluCer and GluSph can directly associate with α-synuclein, enabling the formation of pathogenic aggregates of this protein (36,37). As α-synuclein aggregates are cleared not only by the proteasome but also lysosomes, autophagy/lysosomal pathway (ALP) alterations are believed to contribute to the accumulation and aggregation of α-synuclein (36,38). Our laboratory has recently shown that in nGD human induced pluripotent stem cell (hiPSC)-derived cortical neurons, there is an aberrant activation of mammalian target of rapamycin complex 1 (mTORC1) by elevated GluSph leading to ALP abnormalities and increased neuronal susceptibility (39,40), raising the possibility that a similar mechanism may lead to α-synuclein aggregation in *GBA1*-associated PD. However, although in GD, *GBA1* mutations result in a large increase in GluCer and GluSph levels and clear phenotypic alterations (41), the effects of GCase deficiency in DA neurons from PD patients carrying heterozygote *GBA1* mutations are likely to be more subtle, and thus more difficult to ascribe to the *GBA1* mutation. In addition, the relatively low incidence of PD in GD patients and GD carriers suggests that genetic background, environmental, epigenetic and other factors are important determinants of disease onset and progression. Because of the unknown contribution of these confounding factors, elucidating the specific mechanisms by which mutant *GBA1* increases the risk of PD requires stringent genetic controls.

In this study, we used hiPSC derived from PD patients carrying heterozygote mutations in *GBA1* and gene-edited isogenic controls, to investigate how GCase deficiency affects midbrain DA neurons. We report a mechanism in which GluSph induces the accumulation of pathogenic α-synuclein species through mTOR hyperactivation, and that inhibitors of mTOR or acid ceramidase help to prevent α-synuclein aggregation. Our results suggest that acid ceramidase may be a key player in mutant *GBA1* pathogenesis, and a potential therapeutic target to prevent the deleterious effects of GCase deficiency in *GBA1*-associated neurodegeneration.

## Results

### Mutant DA neurons from PD patients with single-allele *GBA1* mutations have reduced levels of GCase enzyme activity compared with isogenic controls

To examine the effect of mono-allelic *GBA1* mutations on the phenotype of the mutant neurons, we used hiPSC-derived midbrain DA neurons (*GBA1*/PD–DA) from PD patients with heterozygote *GBA1* mutations. hiPSCs from three different PD patients with the genotypes Rec*Nci*I/WT, L444P/WT, N370S/WT and the corresponding gene-edited WT/WT isogenic controls, were differentiated to DA neurons and immunostained with antibodies to the DA marker Tyrosine hydroxylase (TH) and the neuronal marker Tuj1 (Fig. S1A–C) as described in M&M. As shown in Figure S1D, *GBA1*/PD–DA neurons had reduced levels of GCase enzymatic activity compared with the corresponding isogenic controls, as previously reported in PD/DA neurons derived from these hiPSC PD lines (30).

### mTOR inhibition in heterozygote *GBA1*/PD–DA neurons prevents the formation of phosphorylated α-synuclein species

**Figure 1:**
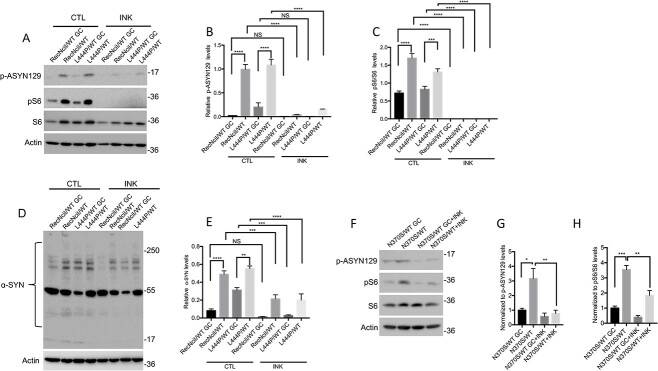
mTOR inhibition in *GBA1*/PD–DA neurons reduces the levels of α-synuclein. (**A**) RecNciI/WT, L444P/WT *GBA1*/PD–DA neurons, and GC controls were either left untreated or were incubated with 50 nm INK128 (INK) for 24 h. Cell lysates were analyzed by WB with antibodies to p-ASYN129, pS6 and S6. The plots at the right of WB represent relative levels of p-ASYN129 (**B**) and pS6 normalized to total S6 (**C**). (**D**) RecNciI/WT, L444P/WT *GBA1*/PD–DA neurons and GC controls were either left untreated or were incubated as in A, shown previously. The detergent-insoluble fractions from cell lysates were analyzed by WB with antibodies to α-synuclein as described in the M&M. (**E**). The plot represents relative levels of α-synuclein in aggregates. (**F**) N370S/WT DA neurons and isogenic controls were treated as described in A, shown previously. Cell lysates were analyzed by WB using antibodies to p-ASYN129, pS6 and S6. The plots at the right of WBs represent relative levels of p-ASYN129 (**G**) and pS6 normalized to total S6 (**H**). Results plotted from three independent experiments (*n* = 3). Error bar represents the mean ± SEM. *P*-values were determined using one-way Analysis of Variance (ANOVA) followed by Bonferroni’s multiple comparisons test. Asterisks indicate the level of statistical significance: ^*^P < 0.05, ^*^^*^P < 0.01, ^*^^*^^*^P < 0.001.

We previously reported that GCase deficiency caused by *GBA1* mutations in nGD cortical neurons, or by pharmacological inhibition of GCase in WT cells, resulted in a block in autophagic flux, and that these alterations were mediated through mTOR hyperactivation (39,40). mTOR is a Ser/Thr kinase that is present in two complexes, mTORC1 and mTORC2. mTORC1 is a nutrient and energy sensor that regulates anabolism, catabolism and the ALP, whereas mTORC2 regulates PI3K, glucose, the cytoskeleton and it is abnormally activated in neoplastic cells (42–45). To examine the effect of heterozygous *GBA1* mutations on mTOR, the ALP and α-synuclein, we carried out Western Blot (WB) and confocal microscopy analysis of mutant versus control DA neurons. As shown in Figure 1A, C, F and H, all three mutants exhibited mTOR hyperactivation, as determined by increased levels of phosphorylation of the mTOR substrate S6 compared with the corresponding gene-edited controls. To examine the functional significance of mTOR hyperactivation in *GBA1*/PD–DA neurons, we tested the effect of mTOR inhibition on α-synuclein aggregation, and the levels of phospho-α-synuclein/Ser129 (p-ASYN129), an α-synuclein species that is present in Lewy bodies (46). WB analysis showed that all three lines of *GBA1*/PD–DA neurons exhibited increased levels of p-ASYN129 (Fig. 1A, B, F and G) compared with isogenic controls. Although RecNciI/WT and L444P/WT *GBA1*/PD–DA neurons also exhibited α-synuclein aggregation compared with isogenic controls (Fig. 1D and E), no increases in α-synuclein aggregation were observed in N370S/WT DA neurons compared with controls (data not shown). Treatment of all three mutant *GBA1*/PD–DA neurons with INK128/TAK228, a catalytic inhibitor of mTOR (40,47) that inhibited S6 phosphorylation (Fig. 1A, C, F and H), prevented the increases in p-ASYN129 and α-synuclein aggregation in the mutant DA neurons described previously (Fig. 1A, B, D, E, F and G).

We conclude from these results that heterozygote *GBA1* mutations cause hyperactivation of the mTORC1 complex, and that this event mediates the formation of pathogenic α-synuclein species in *GBA1*/PD–DA neurons. As mTORC1 is a major regulator of the ALP, these data provide a mechanistic link between mTORC1 deregulation and disruption of the lysosomal functions that are critical for clearance of aggregation-prone proteins and DA neuronal survival. The reversal of α-synuclein accumulation by mTOR inhibitors identifies mTOR as a potential therapeutic target to treat *GBA1*-associated PD.

### Inhibition of GluCer synthase in PD–DA neurons prevents mTOR hyperactivation and p-ASYN129 accumulation

**Figure 2:**
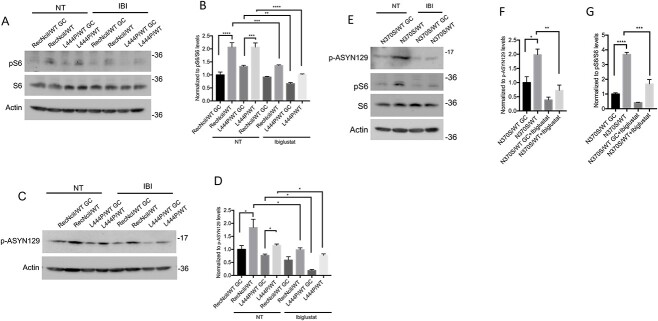
GluCer synthase inhibition in *GBA1*/PD–DA neurons prevents mTOR hyperactivation and elevation of phospho-α-synuclein. (**A**) RecNciI/WT, L444P/WT DA neurons and gene-edited controls were either left untreated or incubated with 1 μm IBI for the last 10 days of DA differentiation. Cell lysates were analyzed by WB with antibodies to p-S6 and S6. (**B**) Plot represents the relative level of p-S6 normalized to total S6. (**C**) RecNciI/WT, L444P/WT DA neurons and isogenic controls were either left untreated or were incubated with IBI as in A, shown previously. Cell lysates were analyzed by WB using antibodies to p-ASYN129. (**D**) The plot represents relative levels of p-ASYN129. (**E**) N370S/WT DA neurons and GC isogenic controls were either left untreated or incubated with IBI as shown previously, and cell lysates were analyzed by WB with antibodies to p-ASYN129, pS6 and S6. The plots represent normalized levels of p-ASYN129 (**F**) and pS6/S6 (**G**). Results are from three independent experiments (*n* = 3). Error bar represents the mean ± SEM. *P*-values were determined using one-way ANOVA followed by Bonferroni’s multiple comparisons test. Asterisks indicate the level of statistical significance: ^*^P % 0.05, ^*^^*^P % 0.01, ^*^^*^^*^P % 0.001, ^*^^*^^*^^*^P % 0.0001.

GluCer synthase catalyzes the formation of GluCer from glucose and ceramide, and its inhibition by Eliglustat is used as substrate reduction therapy (SRT) for type 1 GD, as an alternative to enzyme replacement therapy (48,49). We previously showed that the SRT drugs Eliglustat, and the brain-penetrant GZ161 and Ibiglustat/Venglustat (IBI) (50), are effective in reducing both, GluCer and GluSph accumulation in GD neuronal cells (40). In a recent Phase II clinical trial for type 3 nGD, IBI, in combination with imiglucerase, reduced both GluCer and GluSph levels in plasma and cerebrospinal fluid, and it improved ataxia and neurocognitive deficits (51). To determine whether inhibiting the biosynthesis of GSLs with GluCer synthase inhibitors can prevent the mTOR/α-synuclein alterations caused by GCase deficiency, we treated *GBA1*/PD–DA neurons with IBI for the last 10 days of dopaminergic differentiation. WB analysis showed that this inhibitor prevented mTOR hyperactivation, as determined by decreased levels of pS6 in RecNciI/WT, L444P/WT and N370S/WT DA neurons (Fig. 2A, B, E and G). IBI also reduced p-ASYN129 to isogenic controls levels in all three mutant DA lines (Fig. 2C, D, E and F), but did not significantly decrease α-synuclein aggregation (data not shown).

We conclude from these results that GluCer synthase inhibition by a brain-penetrant SRT drug can prevent mTORC1 hyperactivation and significantly decrease p-ASYN129 levels. However, this treatment was not sufficient to prevent α-synuclein aggregation.

### Inhibition of acid ceramidase in PD–DA neurons is sufficient to prevent the accumulation of pathogenic α-synuclein species

**Figure 3:**
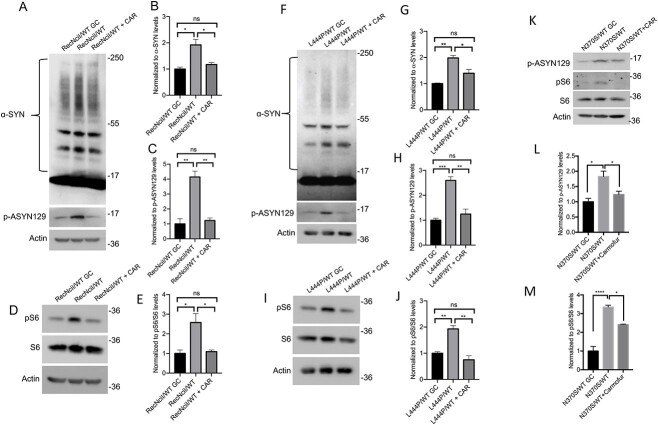
Acid ceramidase inhibition in *GBA1*/PD–DA neurons prevents mTOR hyperactivation and α-synuclein accumulation. RecNciI/WT, L444P/WT and N370S/WT DA neurons and GC isogenic controls were either left untreated or incubated with 1 μm CAR for the last 10 days of DA differentiation as described in the M&M. (**A**) Cell lysates from RecNciI/WT DA neurons and GC controls were analyzed by WB with antibodies to α-synuclein and p-ASYN129. The plots at the right of WB represent relative levels of α-synuclein in aggregates (**B**) and p-ASYN129 levels (**C**) (*n* = 3). (**D**) Aliquots of the RecNciI/WT DA lysates described in A, shown previously, were analyzed by WB using antibodies to pS6 and S6. The plot in (**E**) represents the relative level of p-S6 normalized to total S6. (**F**) Cell lysates from L444P/WT DA neurons and isogenic controls were analyzed by WB with antibodies to α-synuclein and p-ASYN129. The plots at the right of WB represent relative levels of α-synuclein in aggregates (**G**) and p-ASYN129 levels (**H**) (*n* = 3). (**I**) Aliquots of the L444P/WT DA lysates described in F, shown previously, were analyzed by WB using antibodies to pS6 and S6. The plot in (**J**) represents relative level of p-S6 normalized to total S6. (**K**) Cell lysates from N370S/WT DA neurons and isogenic controls were analyzed by WBs with antibodies to p-ASYN129, pS6 and S6. The plot in (**L**) represents relative levels of p-ASYN129. The plot in (**M**) represents the relative level of p-S6 normalized to total S6. Results are from three independent experiments (*n* = 3). Error bar represents the mean ± SEM. *P*-values were determined using one-way ANOVA followed by Bonferroni’s multiple comparisons test. Asterisks indicate the level of statistical significance: ^*^P < 0.05, ^*^^*^P < 0.01, ^*^^*^^*^P < 0.001, ns: non-significant.

As GluCer inhibition prevents the formation of both, GluCer and its deacylated metabolite GluSph (26,52), our results did not allow us to determine which GSL was responsible for the α-synuclein abnormalities we observed. To address this question, we incubated *GBA1*/PD–DA neurons during the last 10 days of dopaminergic differentiation with a low concentration of carmofur (CAR), an acid ceramidase inhibitor (53) that reduces the levels of GluSph in GCase deficient cells (40). As shown in Figures S2A–L and S3A–I, there were no differences in differentiated TH (+) neurons between the mutant and isogenic controls, and treatment with CAR had no effect on the recovery of TH (+) neurons either. As shown in Figure 3D, E, I, J, K and M, CAR treatment of RecNciI/WT and L444P/WT DA neurons prevented mTOR hyperactivation, and reduced p-ASYN129 and α-synuclein aggregation to the levels found in isogenic controls (Fig. 3A, B, C, F, G and H). CAR treatment of N370S/WT DA neurons also prevented mTOR hyperactivation (Fig. 3K and M), and reduced p-ASYN129 levels (Fig. 3K and L), but there was no significant effect on α-synuclein aggregation (data not shown).

**Figure 4:**
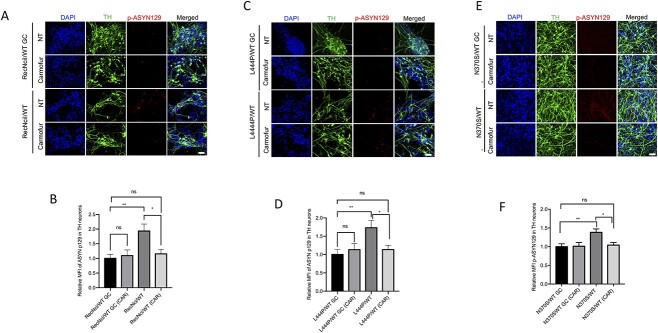
Acid ceramidase inhibition in *GBA1*/PD DA neurons reduces the levels of p-ASYN129. RecNciI/WT, L444P/WT, N370S/WT DA neurons and isogenic controls were either left untreated or incubated with 1 μm CAR during the last 10 days of DA differentiation as indicated. Untreated and treated mutant and control DA neurons were stained with antibodies to TH (green), p-ASYN129 (red) and nuclei were stained with DAPI (blue). The stained samples were analyzed by confocal microscopy as described in M&M (**A**, RecNciI; **C**, L444P; **E**, N370S). The graphs under images represent MFI of p-ASYN129 in TH-positive neurons from the RecNciI (**B**), L444P (**D**) and N370S mutants (**F**). Results are from three independent experiments (*n* = 3). Scale bar, 50 μm. Error bar represents the mean ± SEM. *P*-values were determined using one-way ANOVA followed by Bonferroni’s multiple comparisons test. Asterisks indicate the level of statistical significance: ^*^P < 0.05, ^*^^*^P < 0.01, ns: non-significant.

To further verify these results we performed confocal microscopy analysis on mutant and gene-edited DA neurons (Fig. 4). Image analysis also showed that CAR treatment caused a significant decrease in the levels of p-ASYN129 in DA neurons of all three mutant lines (Fig. 4A–F).

We conclude from these results that because lowering the levels of both, GluCer and GluSph by GluCer synthase inhibition can prevent mTOR hyperactivation and an increase in pathogenic α-synuclein species, inhibiting the enzyme that deacylates GluCer to generate GluSph was sufficient to rescue the abnormal phenotype of mutant DA neurons. We should note that acid ceramidase inhibition was more effective than GluCer synthase inhibition in reducing α-synuclein aggregation in DA neurons harboring severe *GBA1* mutations. Our results suggest that GluSph may be the lipid species largely responsible for these alterations, and that therefore, acid ceramidase is a potential therapeutic target to treat *GBA1*-associated neurodegeneration. Our results are consistent with the idea that persistent GCase deficiency in *GBA1*-associated PD contributes to α-synuclein aggregation through the aberrant activation of mTORC1 by GluSph.

### GCase deficiency in *GBA1*/PD–DA neurons induces an autophagy block that is reversed by inhibitors of mTOR and acid ceramidase

**Figure 5:**
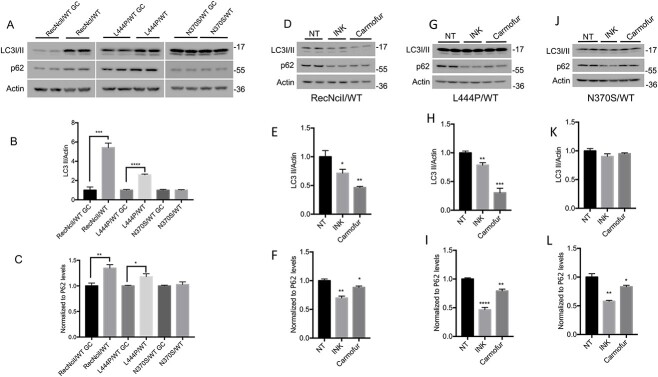
Acid ceramidase inhibition reverts autophagy defects of *GBA1*/PD–DA neurons. (**A**) Cell lysates from RecNciI/WT, L444P/WT, N370S/WT and isogenic control DA neurons, were analyzed by WB using antibodies to LC3 and p62. Plots under the WBs represent normalized levels of LC3-II (**B**) and p62 (**C**). RecNciI/WT (**D**), L444P/WT (**G**) and N370S/WT (**J**) DA neurons were left either untreated or incubated with either 1 nm INK128 or 1 μm CAR during the last 10 days of DA differentiation, and cell lysates were analyzed by WB for levels of LC3 and p62. The graphs under the WBs represent relative levels of LC3 II and p62 in RecNciI/WT (**E, F**), L444P/WT (**H, I**), and N370S/WT (**K, L**) DA neurons. Data were normalized to untreated (NT) controls. Plots represent the results from three independent experiments (*n* = 3). Error bar represents the mean ± SEM. *P*-values were determined using one-way ANOVA followed by Bonferroni’s multiple comparisons test. Asterisks indicate the level of statistical significance: ^*^P < 0.05, ^*^^*^P < 0.01, ^*^^*^^*^P < 0.001, ^*^^*^^*^^*^P < 0.0001.

We previously showed that midbrain DA neurons derived from the *GBA1*/PD hiPSC lines used in this study exhibit an autophagy block (30). As shown in Figure 5A–C, *GBA1*/PD–DA neurons harboring the severe mutations RecNciI and L444P had elevated levels of the autophagy marker LC3-II compared with isogenic controls, whereas no significant differences were detected with N370S. SQSTM1/p62 is an adaptor protein that brings protein aggregates to the autophagosome and is degraded by lysosomes during autophagy (54). As shown in Figure 5A–C, DA neurons from the RecNciI and L444P mutants exhibited increased levels of p62 compared with controls, suggesting a block in the clearance of autophagy substrates. To determine if inhibition of mTOR or acid ceramidase would rescue the autophagy block in *GBA1*/PD–DA neurons, we treated the mutant cells with either INK128 or CAR. As shown in Figure 5D and G incubation of RecNciI/WT and L444P/WT DA neurons with INK128 or CAR reduced the levels of LC3-II and p62 in the mutant cells (Fig. 5E, F, H and I). These inhibitors did not affect LC3-II levels in the N370S/WT mutant (Fig. 5J and K), but p62 levels in the treated N370S/WT neurons were reduced (Fig. 5J and L). Thus, inhibiting mTOR or acid ceramidase was sufficient to ameliorate the autophagic block in *GBA1*/PD–DA neurons.

We conclude from these results that *GBA1*/PD–DA neurons have autophagy defects that may be caused by the deleterious effects of GluSph-dependent mTOR hyperactivation.

### Treatment of gene-edited WT neurons with exogenous GluSph phenocopies the mTOR hyperactivation/α-synuclein phenotype of mutant *GBA1*/PD–DA neurons

The deacylation of GluCer to GluSph results in the formation of a polar, positively charged sphingolipid that exits the lysosome to the cytoplasm (37), where the mTORC1 complex is located. To directly determine if treatment with exogenous GluSph is capable of causing mTORC1 activation and inducing the formation of pathogenic α-synuclein species, we incubated gene-edited WT/WT DA neurons with 2 μm GluSph during the last 10 days of DA differentiation. This treatment caused mTOR hyperactivation as determined by S6 phosphorylation in the gene-edited WT/WT DA neurons derived from the RecNciI/WT (Fig. S4A and B), L444P/WT (Fig. S4C and D) and N370S/WT (Fig. S4E and F) mutants. Significantly, mTORC1 hyperactivation by GluSph was prevented when this treatment was in the presence of the mTOR inhibitor INK128 (Fig. S4A–F).

**Figure 6:**
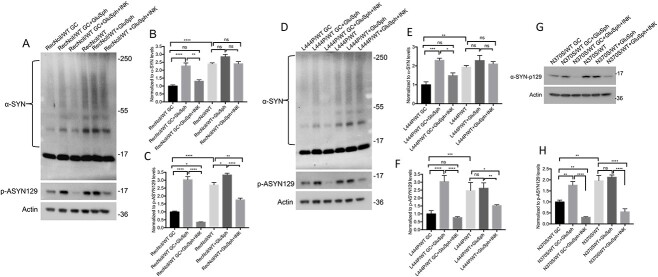
Treatment of gene-edited WT/WT DA neurons with GluSph recapitulates the mTOR-dependent α-synuclein alterations of the parental *GBA1*/PD–DA neurons. (**A**) Gene-edited WT/WT and parental RecNciI/WT DA neurons were either left untreated or incubated with 2 μm GluSph in the presence or absence of 1 nm INK128 during the last 10 days of DA differentiation. Cell lysates were analyzed by WB using antibodies to α-synuclein and p-ASYN129 as described in M&M. The plots represent normalized levels of α-synuclein in aggregates (**B**), and p-ASYN129 levels (**C**). (**D**) Gene-edited WT/WT and parental L444P/WT DA neurons were either left untreated or incubated with GluSph in the presence or absence of INK128 as in A, shown previously. Cell lysates were analyzed by WB using antibodies to α-synuclein and p-ASYN129. The plots represent normalized levels of α-synuclein in aggregates (**E**), and p-ASYN129 levels (**F**). (**G**) Gene-edited WT/WT and parental N370S/WT DA neurons were either left untreated or were incubated with GluSph in the presence or absence of INK128 as described in A, shown previously. Cell lysates were analyzed by WB using antibodies to p-ASYN129. The plot represents normalized levels of p-ASYN129 (**H**) (*n* = 3). Error bar represents the mean ± SEM. *P*-values were determined using one-way ANOVA followed by Bonferroni’s multiple comparisons test. Asterisks indicate the level of statistical significance: ^*^P < 0.05, ^*^^*^P < 0.01, ^*^^*^^*^P < 0.001, ^*^^*^^*^^*^P < 0.0001, ns: non-significant.

**Figure 7:**
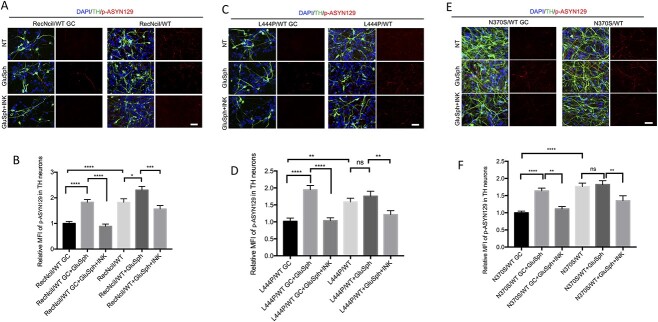
Treatment of WT/WT DA neurons with GluSph recapitulates the mTOR-dependent α-synuclein phenotype of the parental RecNciI/WT, L444P/WT and N370S/WT DA neurons. Gene-edited WT/WT and parental RecNciI/WT (**A**), gene-edited WT/WT and L444P/WT (**C**), and gene-edited WT/WT and N370S/WT (**E**) DA neurons, were either left untreated or incubated with 2 μm GluSph in the presence or absence of 1 nm INK128 during the last 10 days of DA differentiation. Untreated and treated DA neurons were stained for TH (green), p-ASYN129 (red) and nuclei were stained with DAPI (blue) as indicated in the figures. The graphs under images represent MFI of p-ASYN129 in TH-positive neurons from the RecNciI (**B**), L444P (**D**) and N370S (**F**) isogenic pairs (*n* = 3). Scale bar, 50 μm. Error bar represents the mean ± SEM. *P*-values were determined using one-way ANOVA followed by Bonferroni’s multiple comparisons test. Asterisks indicate the level of statistical significance: ^*^P < 0.05, ^*^^*^P < 0.01, ^*^^*^^*^P < 0.001, ^*^^*^^*^^*^P < 0.0001.

We then determined if treatment of gene-edited WT DA neurons with GluSph recapitulated the increase in p-ASYN129 and aggregated α-synuclein observed in the mutant DA neurons. Treatment of gene-edited DA neurons derived from the RecNciI/WT (Fig. 6A–C) and L444P/WT (Fig. 6D–F) mutants with GluSph, induced a 2.5-fold increase in α-synuclein aggregation and a 3-fold increase in p-ASYN129 levels, and these effects were blocked by mTOR inhibition with INK128 (Fig. 6A–F). GluSph also induced an increase in p-ASYN129 in gene-edited neurons derived from N370S/WT cells (Fig. 6G and H), which was prevented by co-incubation with INK128 (Fig. 6G and H). To further verify these results we carried out confocal microscopy analysis on mutant and gene-edited DA neurons (Fig. 7). Image analysis also showed that GluSph treatment caused an increase in p-ASYN129, and that these effects were prevented by INK128 (Fig. 7A–F). It should be noted that pathogenic α-synuclein species were already elevated in *GBA1*/PD–DA neurons, and that further treatment of the mutant cells with GluSph did not cause much increase in α-synuclein (Figs 6 and 7).

**Figure 8:**
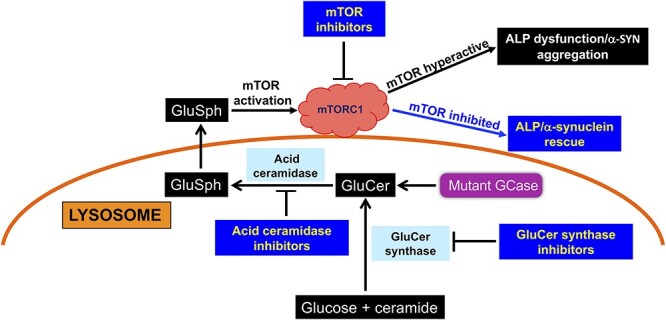
Proposed model of GSL-mediated ALP dysfunction and α-synuclein aggregation in *GBA1*/PD–DA neurons. Mutant GCase causes the accumulation of GluCer in *GBA1*/PD–DA neurons; GluCer is converted to GluSph via an alternate metabolic pathway by the action of lysosomal acid ceramidase. GluSph, which is amphipathic, exits the lysosome into the cytoplasm and activates mTOR complex 1 (mTORC1), leading to ALP dysfunction and α-synuclein aggregation. Inhibitors of GluCer synthase, acid ceramidase and mTOR are able to suppress mTOR hyperactivation and rescue the ALP/α-synuclein phenotype.

We conclude from these results that treatment of multiple gene-edited WT/WT DA neurons with exogenous GluSph phenocopied the mTOR hyperactivation and α-synuclein abnormalities observed in the *GBA1*/PD–DA mutant neurons, and that these effects of GluSph were prevented by mTOR inhibition. Taken together, our results suggest that mTORC1 mediates the ALP/α-synuclein alterations caused by GCase deficiency in heterozygote *GBA1*/PD–DA neurons. A diagram of the model we propose is shown in Figure 8.

## Discussion

In this report we used *GBA1*/PD–DA neurons and isogenic controls to study the specific contribution of mutant *GBA1* to the abnormal phenotype of these neurons. Using this system, we identified an mTORC1-dependent pathogenic mechanism triggered by alternative metabolic conversion of GluCer to GluSph, which interfered with the ALP and clearance of pathogenic α-synuclein species. Our results implicate acid ceramidase as essential for pathogenesis caused by GCase deficiency, and as a potential therapeutic target to treat *GBA1*-associated neurodegeneration.

The presence of two mutant *GBA1* alleles results in GD, and severity of the mutation is a major determinant of disease onset, clinical course and whether there is neurological involvement. However, there is no strict correlation between genotype and clinical phenotype, indicating that other unknown factors contribute to GD. Individuals with heterozygote *GBA1* mutations do not have any symptoms, but they have a 5- to 20-fold increased risk of developing PD (17,55). Because of the low penetration of single allele *GBA1* mutations, the risk of PD is still low, suggesting that these mutations are insufficient to induce PD, and that genetic background, epigenetic, environmental factors and age are important determinants of PD onset and progression. As the unknown contributions to PD of these confounding factors, the pathogenic mechanisms by which heterozygote *GBA1* mutations lead to neurodegeneration are more difficult to study in *GBA1*-associated PD than in nGD, where severe *GBA1* mutations elicit a strong neuronal phenotype. To overcome these limitations we used PD hiPSC-derived DA neurons and the corresponding isogenic controls. These hiPSC pairs were representative of some common *GBA1* mutant genotypes, including the mild N370S, and the severe L444P and RecNciI mutations. Although *GBA1*/PD–DA neurons with all three mutant genotypes exhibited an increase in p-ASYN129, only the RecNciI and L444P variants had an increase in α-synuclein aggregation. As the type of *GBA1* mutation may impact the risk of *GBA1*-associated PD (56), it is possible that the RecNciI and L444P mutations induce a more prominent α-synuclein phenotype that the mild N30S mutation. However, given the lack of genotype–phenotype correlations in GD and PD, further analysis is required to determine the effect of different GBA1 mutations on the risk of PD (57).

There is a substantial body of evidence that in GD and *GBA1*-associated PD, both loss- and gain-of-function mechanisms are involved (40,58–60). However, our data suggest that the phenotypic abnormalities of *GBA1*/PD–DA neurons we observed were caused by a loss of GCase activity. We found that treatment of mutant DA neurons with the GluCer synthase inhibitor IBI rescued the mTOR/ALP/α-synuclein abnormalities of these cells. As this inhibitor lowers the levels of both, GluCer and its metabolite GluSph, we then used an acid ceramidase inhibitor to further examine which of these two GSLs was largely responsible for the deleterious effects of GCase deficiency in the mutant neurons. Our results clearly implicated GluSph as part of the mechanism by which GCase deficiency deregulates *GBA1*/PD–DA neurons.

GluSph is barely detectable in normal cells, but in nGD patient brains there is up to a 500-fold elevation of this lipid (10,11,24), and there are also large accumulations of GluSph in plasma, even in GD1, as this polar lipid leaves the lysosomal compartment into the cytoplasm and extracellular space (25,37). There are also reports of increased levels of GluCer and GluSph in brain regions (including the substantia nigra and hippocampus) from sporadic PD patients and during aging (61–63), but it should be noted that other groups have not shown GSL accumulation in brains from *GBA1*-associated PD. Given that the levels of GluCer and GluSph are much lower in *GBA1* heterozygotes and sporadic PD than in GD (64), overall sphingolipid alterations are less likely to be detected. Nonetheless, our results in the current cellular study *in vitro*, lend support to the idea that even slow but persistent generation of GSLs over a period of decades may contribute to the onset and progression of *GBA1*-associated PD.

Our analysis showed that *GBA1*/PD–DA neurons exhibited mTOR hyperactivity, which in turn deregulated the ALP, leading to the accumulation of pathogenic α-synuclein species. Several lines of evidence support a mechanism in which these abnormalities result from aberrant activation of mTORC1 by excess formation of GluSph. First, treatment of the mutant DA neurons with mTOR inhibitors restored ALP function and reduced α-synuclein to isogenic control levels, suggesting that the abnormal α-synuclein phenotype was directly related to mTORC1 hyperactivity. Second, treatment of *GBA1*/PD–DA neurons with an acid ceramidase inhibitor was sufficient to prevent mTOR hyperactivation, restore autophagic flux and reduce α-synuclein accumulation and aggregation. These results suggest that in the mutant DA neurons, GluSph may directly or indirectly activate mTORC1. This conclusion is supported by the observation that treatment of gene-edited WT/WT DA neurons with exogenous GluSph phenocopied the mTOR and ALP/α-synuclein alterations of *GBA1*/PD–DA neurons. Moreover, co-incubation of WT/WT control DA neurons with GluSph and INK128 prevented α-synuclein accumulation and aggregation, suggesting that the phenotypic abnormalities induced by exogenous GluSph were also mediated by mTORC1 hyperactivity. These results are consistent with the idea that GluSph deregulates the ALP, interfering with its ability to clear pathogenic α-synuclein species. A key role of GluSph and acid ceramidase in *GBA1*-associated neuropathology is also supported by studies in a relevant mouse model of *GBA1*-associated PD (24,37). Taguchi *et al*. showed that CAR significantly decreased the levels of oligomeric precursors of aggregated α-synuclein in brain, suggesting that *in vivo*, GluSph is likely to be a key neurotoxic metabolite responsible for *GBA1*-associated neuropathology (24,61). Additionally, other studies have shown that CAR treatment of HEK293-FT cells deficient in GCase and *GBA1*/PD dopamine neurons resulted in decreased oxidized α-synuclein (22).

Although our results suggest a GluSph/mTOR-mediated mechanism leading to ALP/α-synuclein abnormalities, other mechanisms have also been reported. It has been proposed that GluCer and GluSph interact directly with α-synuclein, promoting its aggregation (37,65–68). Also, a decreased ability of mutant GCase to break down GluCer to ceramide, which can result in decreased levels of ceramide, may also contribute to *GBA1*-associated PD (22,69,70). As GCase deficiency results in downstream abnormalities in other lipids including phosphatidic acid, phosphatidylethanolamine, plasmal-ogenphosphatidylethanolamine (PEp), acyl phosphatidylglycerol, lactosylceramide and gangliosides (63,71–73), additional studies are required to delineate their contribution to *GBA1* dysfunction and pathogenesis in PD and Lewy Body Dementia (LBD) syndromes (74–76). In addition, GluCer is a constituent of lipid bilayers, and alterations in GSLs as a consequence of GCase deficiency may affect normal membrane functions, as well as trafficking and fusion between vesicles and organelles including lysosomes (77–80). Although our data and many reports in the literature support a model in which loss of GCase enzymatic activity is central to *GBA1*-associated neurodegeneration, there is also substantial evidence for upstream biological mechanisms, in which endoplasmic reticulum-associated degradation and the unfolded protein response induced by mutant GCase disrupts proteostatic and membrane transport mechanisms that are essential for neuronal survival (59,60). In addition, work with fibroblasts from patients with sporadic PD provide evidence of systemic involvement of *GBA1* dysfunction; in that case, the loss of *GBA1* activity was caused primarily by loss of transport of *GBA1* by LIMP2 from the endoplasmic reticulum (ER) to the lysosome (81).

One of the most interesting findings of our study is that acid ceramidase may play a key role in *GBA1*-associated neurodegeneration, suggesting that this enzyme may be a therapeutic target for alternative or combination SRT. This conclusion is supported by reports that the elevation of sphingoid bases leads to neurodegeneration in other sphingolipidoses as well (26). For instance, Krabbe is a fatal demyelinating disease caused by galactosylceramidase deficiency. In this disorder, the lipid that accumulates is galactosylsphingosine (GalSph), which is generated by the action of acid ceramidase (82,83). Li *et al*. showed that crossing of Krabbe (Twitcher) mice with *Asah1*/acid ceramidase-deficient mice (Farber mice) prevented accumulation of GalSph and cured the Krabbe mice (82). Additionally, intraperitoneal injection of CAR into Krabbe mice decreased the levels of GalSph and extended their lifespan. Thus, acid ceramidase is likely to be an important therapeutic target in sphingolipidoses where there is an accumulation of lyso-glycosphingolipids including Gaucher, Krabbe and Fabry diseases (26,84).

In summary, the results presented here provide compelling evidence for a mechanism in which persistent GCase deficiency results in steady-state generation of GluSph in mutant DA neurons. In turn, aberrant mTORC1 activation by this lipid suppresses critical ALP functions including the ability of DA neurons to clear accumulating α-synuclein species. This GluSph/mTORC1/ALP pathogenic mechanism is similar to the one we recently reported in nGD neurons (40). Thus, although nGD and PD are different diseases, the inability to break down GluCer appears be a common denominator in the pathogenic cascade of metabolic and molecular events leading to *GBA1*-associated neurodegeneration. Finally, our results suggest that acid ceramidase plays a critical role in the pathogenesis of *GBA1*-associated PD, identifying this enzyme as a potential therapeutic target for the treatment of *GBA1*-associated neurodegeneration and other disorders where there is an elevation of deacylated GSLs.

## Materials and Methods

### Maintenance and differentiation of hiPSCs to DA neurons

The hiPSC lines from a healthy WT/WT control, and from PD patients carrying heterozygous *GBA1* mutations (RecNcil/WT, L444P/WT, N370S/WT) and the corresponding gene-corrected (GC) WT/WT isogenic controls were previously described (30,85). These lines are listed in Supplementary Material, Table S1. The hiPSCs were cultured using standard protocols on inactivated mouse embryonic fibroblasts. Differentiation of hiPSC to dopamine neurons was carried out as described (86). Single cell hiPSCs were cultured on matrigel-coated plates at a density of 40 000 cells/cm^2^ in Serum replacement media containing growth factors and small molecules including FGF8a (100 ng/ml), SHH C25II (100 ng/ml), LDN193189 (100 nm), SB431542 (10 μm), CHIR99021 (3 μm) and Purmorphamine (2 μm) for 5 days. The next 6 days, cells were maintained in neurobasal medium containing B27 minus vitamin A, N2 supplement and LDN193189 and CHIR99021. Then single cell suspensions were seeded at a density of 400 000/cm^2^ on polyornithine- and laminin-coated plates in neurobasal media containing B27 minus Vitamin A, Brain-Derived Neurotrophic Factor (BDNF) (20 ng/ml), Glial cell line-derived neurotrophic factor (GDNF) (20 ng/ml), Transforming growth factor beta (TGFβ) (1 ng/ml) ascorbic acid (0.2 mm), cyclic AMP (cAMP) (0.5 mm) and DAPT (10 μm) until maturation for ⁓60 days.

### Immunocytochemistry

hiPSC-DA neurons were grown on polyornithine- and laminin-coated glass coverslips for microscopy as follows**.** Glass coverslips were coated with polyornithine at 100 μg/ml diluted in sterile water for a minimum of 3 h at room temperature, followed by three washes in sterile water. Laminin was added to the precoated glass coverslips at 5 μg/ml diluted with sterile phosphate-buffered saline (PBS) and incubated for a minimum of 8 h in a 5% CO_2_ incubator. Laminin was removed and DA differentiation was carried out in the coated coverslips. After differentiation, neurons were fixed with 4% paraformaldehyde (Santa Cruz) for 15 min at room temperature followed by three washes with 1× PBS. The neurons were permeabilized with 0.3% (Vol/Vol) Triton X-100 for 15 min and blocked for 1 h in 5% (Vol/Vol) normal goat serum in PBS. This was followed by incubation with the indicated primary antibodies diluted in 5% goat serum/PBS overnight at 4°C. Following three washes with 1× PBS, cells were incubated with secondary antibody diluted at 1:1000 in 5% goat serum/PBS for 1 h in the dark at room temperature. The secondary antibody was removed, followed by three washes with 1× PBS. Cover slips were mounted with DAPI-containing mounting media on glass slides and kept in the dark. After 24 h, mounted cover slips were ready for confocal imaging.

### Microscopy and imaging

Immunofluorescence images were captured using a Nikon A1 confocal laser scanning microscope under 20× or 60× oil objectives. The excitation wavelengths used were 405, 488 and 561 nm for blue, green and red fluorophores, respectively. Neuronal images were acquired as Z-stacks. Identical pixel acquisition settings were used for all experiments. Further image processing and analysis was done using Fiji or Image J software (https://imagej.nih.gov/ij). Fluorescence intensity of the respective signals was obtained from 150–200 TH positive neurons from at least three independent experiments. The mean fluorescence intensity (MFI) was calculated accordingly.

### GCase assay

The assay for GCase enzymatic activity in intact cells was carried out as described (41). DA progenitor cells were plated in 96-well plates at a density of 400 000/cm^2^ for DA differentiation. At day 60 of differentiation, the medium was removed and neurons were washed with PBS. The assay reaction was started by the addition of 50 μl of 2.5 mm 4-methylumbelliferyl β-D-glucopyranoside (MUG) (Sigma) in 0.2 m acetate buffer (pH 4.0) to each well. Plates were incubated at 37**°**C for 2 h and the reaction was stopped by the addition of 150 μl of 0.2 m glycine buffer (pH 10.8) to each well. Released 4-methylumbelliferone was measured using a SpectraMax Gemini plate reader (Molecular Device, Sunnyvale, CA) (excitation 365 nm, emission 445 nm). Conduritol B epoxide was added at 1 mm to replicate wells for the duration of the assay, to control for non-GCase enzymatic activity.

### Chemical reagents and treatments

CAR (14243) was from Cayman Chemical; INK128 was from Selleck Chemicals; Glucosyl(ß) Sphingosine (d18:1) (860535P) was from Avanti Polar Lipids. All of the reagents used in this study are listed in Supplementary Material, Table S2.

The drugs were added to the culture medium directly and were replenished with every media change. For acid ceramidase and mTOR inhibition, neuronal cells were incubated with CAR and INK128 at final concentration of 1 μm and 1 nm, respectively, during the last 10 days of DA differentiation and were replenished with every media change. For exogenous GluSph treatment, differentiated neurons were incubated with a final concentration of 2 μm during the last 10 days of DA differentiation and were replenished with every media change.

### Immunoblot analysis

For immunoblot analysis, differentiated DA neurons were scraped in 1× PBS on ice and collected by centrifugation. Cell lysates for nonionic detergent-soluble and detergent-insoluble fractions were made by homogenization of the cell pellet in lysis buffer containing 1× PBS, 1% Triton X100 (Vol/Vol) and phosphatase/protease inhibitor mixture (Cell Signaling Technology). Cell pellets were briefly vortexed three times at 5 min intervals and kept on ice. The homogenates were centrifuged for 30 min at 4°C, 14 000 rpm, and the supernatant was collected as the soluble fraction. The leftover pellet was washed with ice-cold PBS and sonicated five times for 10 s at 2 s intervals on ice in a lysis buffer containing 5% SDS. The homogenate was centrifuged for 30 min at 4°C, 14 000 rpm, and the resulting supernatant (nonionic detergent-insoluble fraction) was collected to determine the extent of α-synuclein aggregation. The samples were analyzed on 4–20% Tris-Glycine gradient SDS-PAGE gels (Thermofisher). Electrophoresis was followed by protein transfer onto nitrocellulose membranes. Membranes were blocked with 5% (WT/Vol) non-fat dry milk in Tris-buffered saline with 1% Tween-20 (TBS-T) and incubated with the indicated primary antibodies for 2–3 h at room temperature or overnight at 4°C. After horseradish peroxidase-conjugated secondary antibody incubation, the membranes were developed with SuperSignal West Femto Maximum Sensitivity Substrate (ThermoFisher Scientific), and imaged using the Chemidoc system and Imagelab software (BioRad). Densitometry analysis was done using Image J software.

### Mean fluorescence intensity quantification

Confocal Z stacked images were analyzed for mean fluorescent Intensity (MFI). For fluorescence intensity measurements, regions of interest (ROI) were measured from groups of 15–30 TH-positive neurons from three independent experiments. Manually ROI were drawn on TH-positive neurons, and MFI was analyzed with Image J software (version 2.0.0-rc-68/1.52e, open-source platform for biological image analysis) for MAC OS X, using the Red, Green and Blue (RGB) measure function (87).

### Cell counting

Images were acquired for TUJ/TH/DAPI staining using a confocal laser-scanning microscope under a 20× or 60× oil objective from three coverslips for each condition. A total of 450–550 cells were counted for each condition using the cell counter function in Image J.

### Quantification and statistical analysis

Statistical analysis was performed using GraphPad Prism software version 7.0a. The n—number indicates the number of independent experiments. Statistical analyses were performed using one-way ANOVA followed by Bonferroni’s multiple comparisons test, as indicated in the figure legends. All data sets were selected for analysis. Multiple comparisons of the mean of preselected pairs of data sets were analyzed using Bonferroni post hoc tests. In each figure, asterisks indicate the level of statistical significance: ^*^P ≤ 0.05, ^*^^*^P ≤ 0.01, ^*^^*^^*^P ≤ 0.001, ^*^^*^^*^^*^P ≤ 0.0001 and ns: non-significant. Results are expressed as mean ± Standard error of mean (SEM).

## Supplementary Material

Figure_S1_ddad025

Figure_S2_ddad025

Figure_S3_ddad025

Figure_S4_ddad025

Supplemental_Figure_Legends_ddad025

Supplementary_Table_1_ddad025

Supplementary_Table_2_ddad025

## Data Availability

All the data used in this study are available upon request.
