# Supplementary figures and images for: Acid ceramidase involved in pathogenic cascade leading to accumulation of α-synuclein in iPSC model of GBA1-associated Parkinson’s disease
Source: Hum Mol Genet. 2023 Feb 8;32(11):1888–900. doi: 10.1093/hmg/ddad025 (PMC10196677; doi:10.1093/hmg/ddad025)

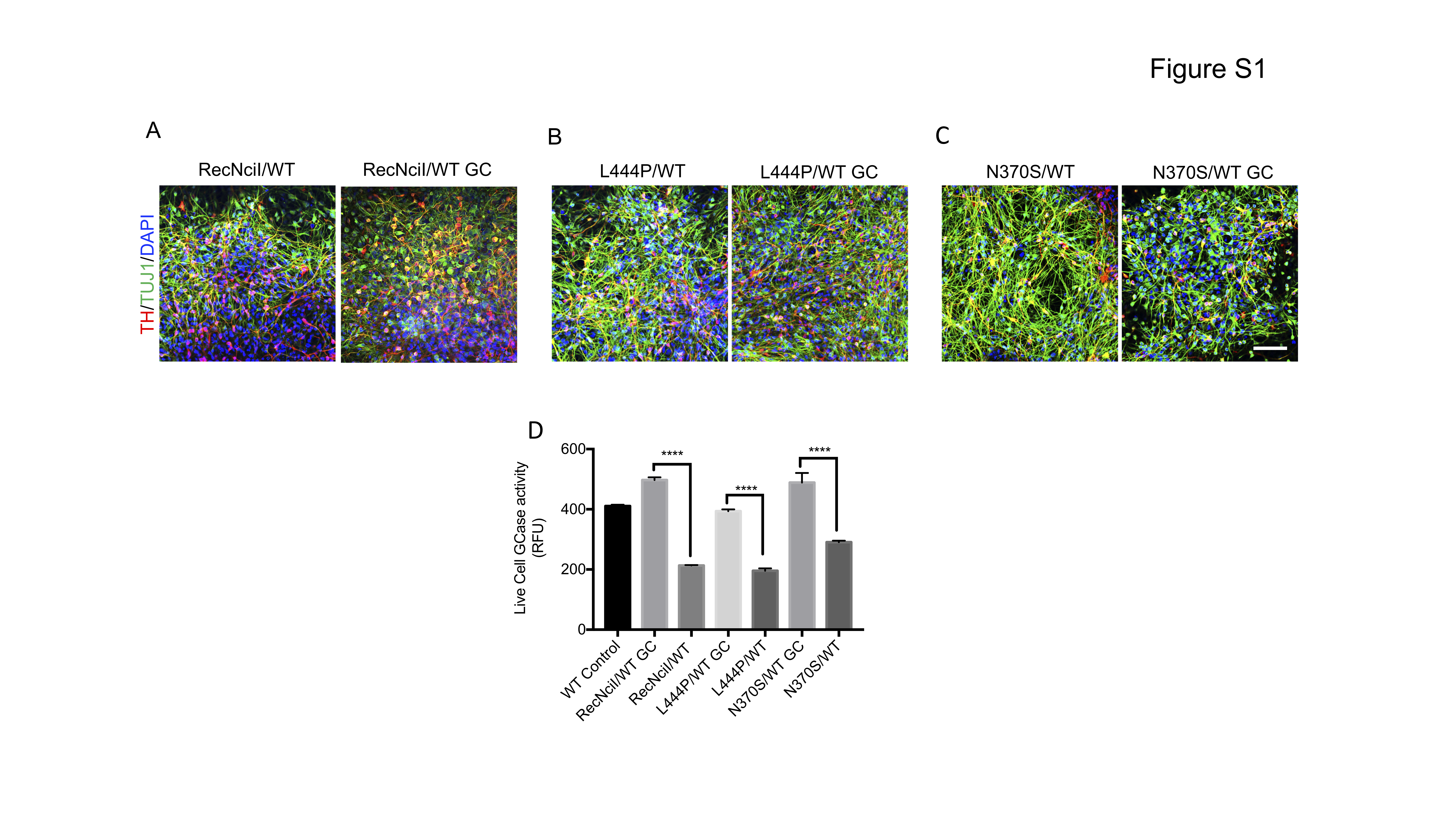

Supplement: Figure_S1_ddad025 [file figure_s1_ddad025.zip › Figure_S1_ddac025.tiff]

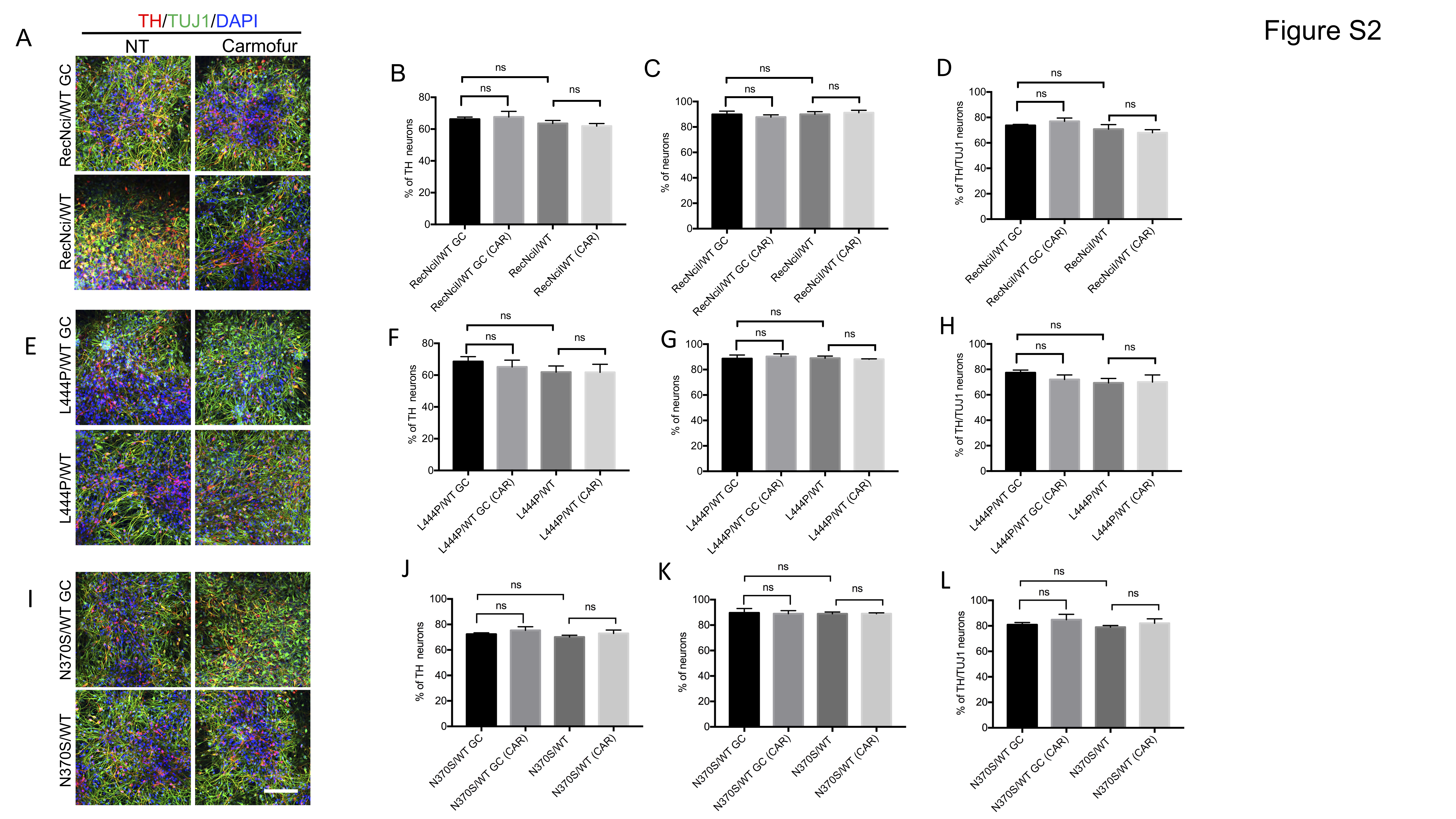

Supplement: Figure_S2_ddad025 [file figure_s2_ddad025.zip › Figure_S2_ddac025.tiff]

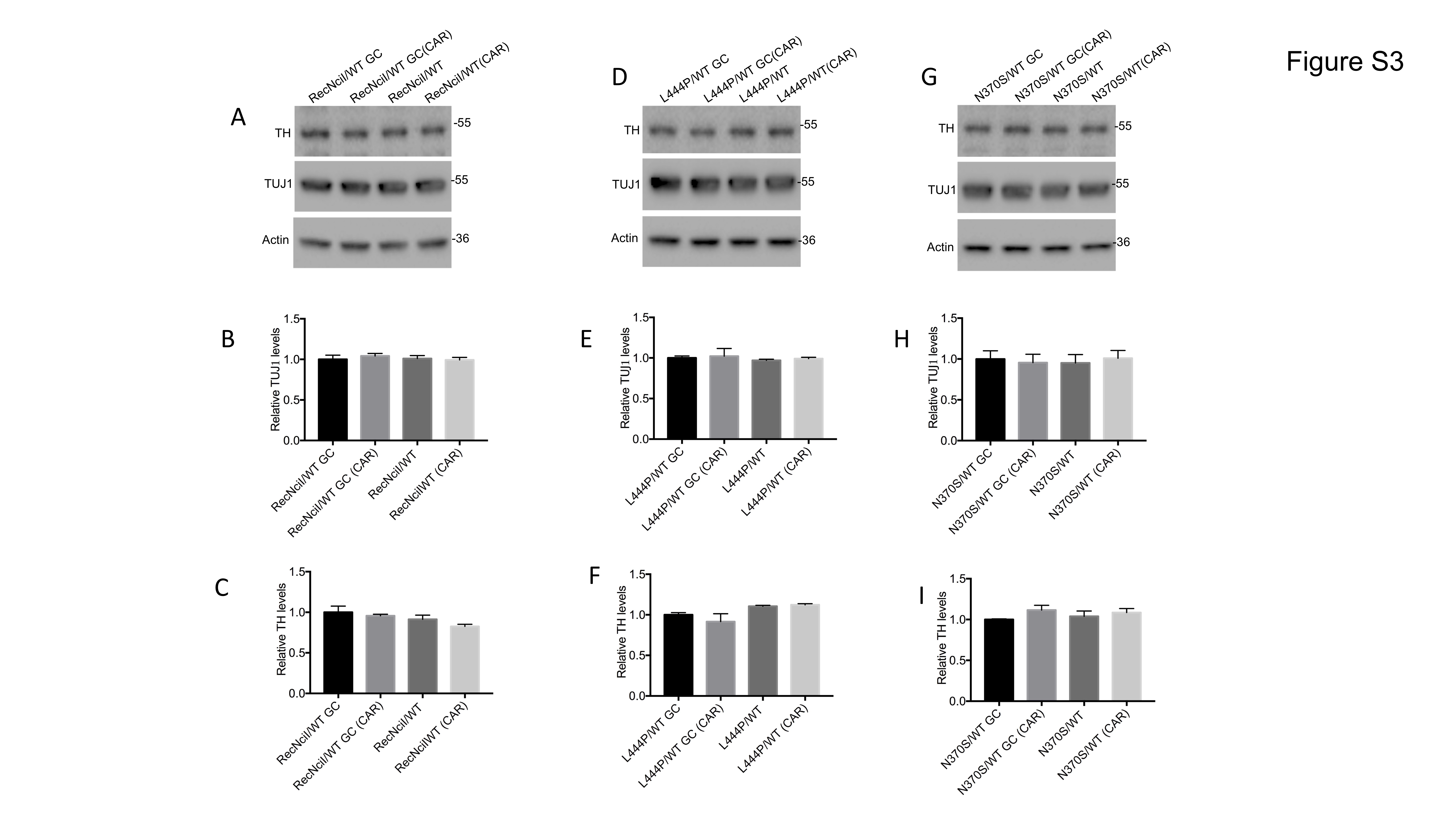

Supplement: Figure_S3_ddad025 [file figure_s3_ddad025.zip › Figure_S3_ddac025.tiff]

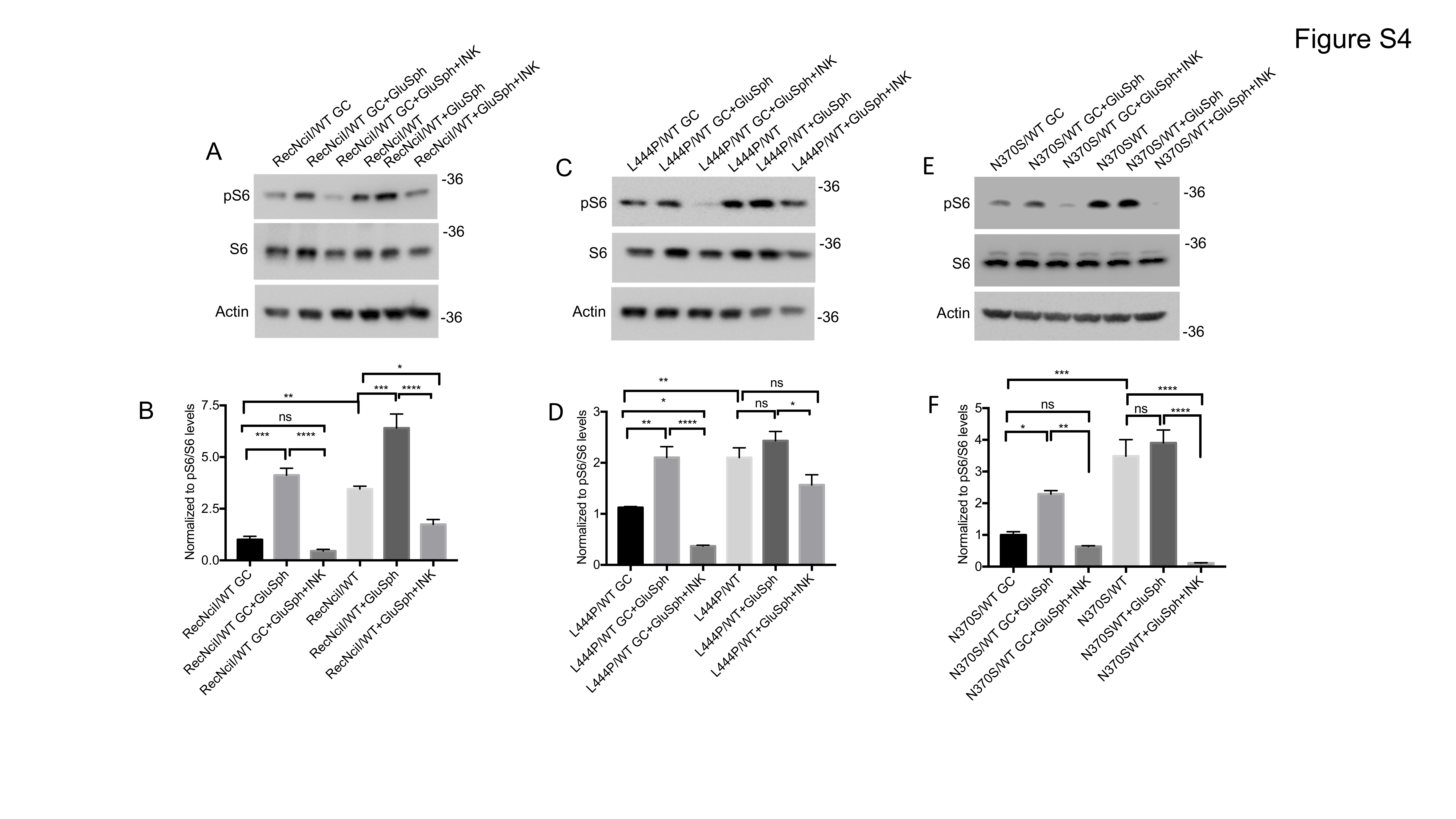

Supplement: Figure_S4_ddad025 [file figure_s4_ddad025.zip › Figure_S4_ddac025.tiff]
