# Supplementary material for: Acid ceramidase involved in pathogenic cascade leading to accumulation of α-synuclein in iPSC model of GBA1-associated Parkinson’s disease
Source: Hum Mol Genet. 2023 Feb 8;32(11):1888–900. doi: 10.1093/hmg/ddad025 (PMC10196677; doi:10.1093/hmg/ddad025)
Supplement: Supplemental_Figure_Legends_ddad025 [file supplemental_figure_legends_ddad025.docx]

**Supplemental Figure 1. Differentiation of hiPSC harboring heterozygote *GBA1* mutations to midbrain dopaminergic neurons.** RecNciI/WT **(A),** L444P/WT **(B),** N370S/WT **(C)** *GBA1*/PD hiPSC, and the corresponding gene-corrected (GC) controls were differentiated to DA neurons and then stained with antibodies to the DA marker Tyrosine hydroxylase (TH, red) and the neuronal marker Tuj1 (green); Nuclei were stained with DAPI (blue). Scale bar, 100 µm. **(D)** WT/WT control, RecNciI/WT, L444P/WT**,** N370S/WT DA neurons, and the 3 corresponding gene-edited isogenic controls were assayed for GCase enzymatic activity as described in the M&M. The plot represents GCase activity in mutant vs. control DA neurons quantitated from three independent experiments (n=3). Error bar represents the mean ± SEM. p-values were determined using one-way ANOVA followed by Bonferroni’s multiple comparisons test. Asterisks indicate the level of statistical significance: *p < 0.05, **p < 0.01, ***p < 0.001, ****p < 0.0001. RFU, relative fluorescence units.

**Supplemental Figure 2. Carmofur treatment of *GBA1*/PD-DA neurons does not affect recovery of DA neurons.** RecNciI/WT **(A)**, L444P/WT **(E)**, N370S/WT **(I)** DA neurons, and the corresponding isogenic GC controls were either left untreated or were incubated with 1 μM carmofur (CAR) during the last 10 days of DA differentiation. Untreated and treated DA neurons were stained for TH (red) and Tuj1 (green); Nuclei were stained with DAPI (blue). The plots represent the % of neurons stained for TH **(B, F, J),** Tuj1**(C, G, K)**, and the normalized levels of TH/Tuj1 **(D, H, L)** neurons from the mutant DA neurons and isogenic controls. The graphs were from three independent experiments (n=3). Scale bar, 100 µm. Error bar represents mean ± SEM. p-values were determined using one-way ANOVA followed by Bonferroni’s multiple comparisons test, ns: non-significant.

**Supplemental Figure 3. Carmofur treatment of *GBA1*/PD-DA neurons does not affect recovery of DA neurons.**  Gene-edited WT/WT and parental RecNciI/WT **(A)**, gene-edited and parental L444P/WT **(D)**, and gene-edited and parental N370S/WT **(G**) DA neurons, were either left untreated or incubated with 1 μM carmofur (CAR) during the last 10 days of DA differentiation. Cell lysates from the indicated mutant and isogenic controls were analyzed by WB using antibodies to TH and TUJ1. The plots under the blots **(B, E and H)** represent relative levels of TUJ1 neurons, and **(C, F and I)** represent relative levels of normalized TH neurons from the mutant *GBA1*/PD-DA neurons and isogenic controls. The results are quantitated from three independent experiments (n=3). Error bar represents the mean ± SEM. p-values were determined using one-way ANOVA followed by Bonferroni’s multiple comparisons test.

**Supplemental Figure 4. mTOR inhibition in isogenic WT/WT DA neurons blocks S6 phosphorylation induced by GluSph.** Gene-edited WT/WT and parental RecNciI/WT **(A)**, gene-edited WT/WT and parental L444P/WT **(C)**, and gene-edited WT/WT and parental N370S/WT **(E**) DA neurons, were either left untreated or incubated with 2 μM GluSph in the presence or absence of 1 nM INK128 during the last 10 days of DA differentiation. Cell lysates from the mutant and corresponding isogenic controls were analyzed by WB using antibodies to pS6 and S6. The plots under the blots **(B, D, and F)** represent levels of pS6 normalized to S6. Plots represent the results from three independent experiments (n=3). Error bar represents the mean ± SEM. p-values were determined using one-way ANOVA followed by Bonferroni’s multiple comparisons test. Asterisks indicate the level of statistical significance: *p < 0.05, **p < 0.01, ***p < 0.001, ****p < 0.0001, ns: non-significant.
