# Supplementary material for: Acid ceramidase involved in pathogenic cascade leading to accumulation of α-synuclein in iPSC model of GBA1-associated Parkinson’s disease
Source: Hum Mol Genet. 2023 Feb 8;32(11):1888–900. doi: 10.1093/hmg/ddad025 (PMC10196677; doi:10.1093/hmg/ddad025)
Supplement: Supplementary_Table_1_ddad025 [file supplementary_table_1_ddad025.doc]

**Supplementary Table 1. Genotypes of hiPSC lines used in the study.**

| **Genotype** | **Gene-corrected (GC) genotype** |
| --- | --- |
| WT/WT Control |  |
| RecNciI/WT | WT/WT |
| L444P/WT | WT/WT |
| N370S/WT | WT/WT |
