# Supplementary material for: Acid ceramidase involved in pathogenic cascade leading to accumulation of α-synuclein in iPSC model of GBA1-associated Parkinson’s disease
Source: Hum Mol Genet. 2023 Feb 8;32(11):1888–900. doi: 10.1093/hmg/ddad025 (PMC10196677; doi:10.1093/hmg/ddad025)
Supplement: Supplementary_Table_2_ddad025 [file supplementary_table_2_ddad025.doc]

**Supplementary Table 2. List of reagents.**

| **REAGENTS AND RESOURCES** | **SOURCE** | **IDENTIFIER** |
| --- | --- | --- |
| **Antibodies** | | |
| TH | Millipore | AB152 |
| TUJ1 | Biolegend | 801202 |
| LAMP1 | Cell Signalling | 15665S |
| Asyn p 129 | Millipore | MABN826 |
| Asyn p 129 | Cell Signalling | 23706 |
| ASYN | BD | 610787 |
| pS6 | Cell Signalling | 4856s |
| pS6 | Cell Signalling | 62016S |
| S6 | Cell Signalling | 2217s |
| GBA1 | Sigma | WH0002629M1 |
| Actin | Sigma | A3854 |
| LC3 | Cell Signalling | 4599 |
| P62 | Cell Signalling | 8025 |
| Anti-Mouse IgG | Thermo Fisher | A-11029 |
| Anti-Rabbit IgG | Thermo Fisher | A-11036 |
| Anti-mouse IgG, HRP | Cell Signalling | 7076S |
| Anti-rabbit IgG, HRP | Cell Signalling | 7074S |
|  |  |  |
| **Chemicals, Peptides, and Recombinant Proteins** | | |
| DMEM F12 | Thermo Fisher | 11-330-057 |
| DMEM | Thermo Fisher | 11-965-118 |
| Neurobasal | Thermo Fisher | 21103049 |
| Glutamax | Thermo Fisher | 35050061 |
| Penicillin-Streptomycin | Thermo Fisher | 15140-122 |
| KnockOut™ Serum Replacement | Thermo Fisher | 10828028 |
| FBS | Hyclone | SH30071.03 |
| MEM Non-Essential Amino Acids | Thermo Fisher | 11140050 |
| Collagenase | Thermo Fisher | 17104019 |
| 2-Mercaptoethanol | Thermo Fisher | 21985023 |
| DMSO | Sigma | D2650 |
| Accutase | Thermo Fisher | A1110501 |
| Matrigel | CORNING | 354230 |
| Mitomycin C | Sigma | M4287 |
| Laminin | Sigma | L2020 |
| N-2 Supplement | Thermofisher | 17502048 |
| B-27 Supplement | Thermofisher | 1103049 |
| bFGF | Peprotech | 00-18B |
| BDNF | R&D System | 248-BDB-050 |
| GDNF | R&D System | 212-GD-050 |
| TGFβ3 | R&D System | 243-B3-002 |
| SHH C25II | R&D System | 464-SH-025 |
| FGF-8 | R&D System | 423-F8-025 |
| dbcAMP | Sigma | D0627 |
| Ascorbic acid | Sigma | A4034 |
| LDN193189 | Stemgent | 04-0074-02 |
| Purmorphamine | Stemgent | 04-0009 |
| DAPT | Stemgent | 04-0041 |
| CHIR | Stemgent | 04-0004-02 |
| SB432542 | Stemgent | 04-0010-05 |
| Polyornithine | Sigma | P3655 |
| Y-27632 | Sigma | Y0503 |
| Normal Goat Serum | Cell Signalling | 5425S |
| Novex™ 4-20% Tris-Glycine Mini Gels | Thermo Fisher | XP04205BOX |
| Protease/Phosphatase Inhibitor Cocktail (100X) | Thermo Fisher | 5872S |
| Nitrocellulose Membrane | Thermo Fisher | 88018 |
| Prestained Protein Ladder | Thermo fisher | 26619 |
| Glucosylsphingosine | AVANTI polar lipids | 860535 |
| 4-Methylumbelliferyl β-D-glucopyranoside | Sigma | **M3633** |
| INK-128 | Selleck Chemicals | 1224844-38-5 |
| Carmofur | Cayman Chemical | 14243 |
| Ibiglustat | P14969 | AstaTech |
| Conduritol β-epoxide | C5424 | **Sigma** |
|  |  |  |
| **Software and Algorithms** | | |
| GraphPad Prism | GraphPad Software | N/A |
| Fiji | NIH | N/A |
| ImageJ | NIH | N/A |
| Image Lab | Biorad | N/A |
